# Evaluation of Nasal Reconstruction With Local Flaps Using the Translated Version of the FACE-Q Scale in a Local Language

**DOI:** 10.7759/cureus.80409

**Published:** 2025-03-11

**Authors:** Arnab Sarkar, Umesh Kumar, Suman Babu Gottam, Yasharth Sharma, Nikhil Prasad

**Affiliations:** 1 Plastic Surgery, Parul Institute of Medical Sciences and Research, Parul University, Vadodara, IND; 2 Plastic and Reconstructive Surgery, Institute of Medical Sciences, Banaras Hindu University, Varanasi, IND; 3 Plastic and Reconstructive Surgery, Moti Lal Nehru (MLN) Medical College, Prayagraj, IND

**Keywords:** face-q scale, nasal reconstruction, nasolabial flap, paramedian forehead flap, rhinoplasty

## Abstract

Introduction

Attaining a nasal reconstruction that is functionally and aesthetically pleasing is a challenge to many reconstructive surgeons worldwide. Since the concept of the ‘subunit principle’ was laid down, many advancements have been devised in nasal reconstruction, but no proper evidence-based data is yet present in the literature regarding the satisfaction amongst patients after nasal reconstruction. In this study, we have evaluated the outcome after nasal reconstruction using a local language translation of FACE-Q scales.

Materials and methods

A total of 23 patients were operated on for nasal reconstruction in one or two stages during the period from September 2021 to December 2022, after assessing the cause, the subunit involved, and the type of nasal defect. The FACE-Q questionnaire was administered preoperatively and six months postoperatively to assess the satisfaction with outcomes among patients.

Results

Fifty-one nasal defects were reconstructed in 23 patients, out of which the nasal tip was the most commonly involved subunit. The defects were resurfaced with full-thickness skin grafts, V-Y advancements, deltopectoral flaps, nasolabial flaps, axial frontonasal flaps, and paramedian forehead flaps. Almost all the patients showed a significant improvement in their FACE-Q postoperative scales after the surgeries.

Conclusion

Nasal reconstruction, owing to its complex anatomy, has always been a subject of discussion. Comprehensive information about the outcome of nasal reconstruction can be measured using the FACE-Q scale, which is a patient-reported outcome assessment. The evaluation will always help the surgeon in regularly assessing and improving the outcome of surgery.

## Introduction

Nasal reconstruction is a challenging task due to its aesthetic and functional importance. An aesthetically pleasing nose boosts the confidence of an individual and psychologically impacts his/her quality of life (QoL) in society. The quest for nasal reconstruction has been noted since the time of Sushruta, who, around 600 to 700 BC, outlined a cheek flap for its reconstruction [1]. Since then, nasal reconstruction has evolved with the aim to restore a near-normal appearance and function of the nose. In 1985, Burget and Menick laid down the ‘subunit principle’ by dividing the nose into aesthetic subunits and devising reconstruction techniques particular to each subunit [2]. Regardless of all the advancements, there remains a lacuna in the literature regarding an evidence-based approach to nasal reconstruction.

Evaluation of patient satisfaction and QoL (quality of life) after reconstructive surgery is becoming crucial today. Proper, quantifiable outcome analysis helps surgeons individualize reconstructive options catering to a specific type of defect. The FACE-Q module developed by Klassen et al., is a patient-reported outcome (PRO) tool, comprising different appraisal scales for different areas of the face. The module uses generalized questions evaluating the patient’s satisfaction with respect to aesthetic and functional outcomes before and after surgery and any adverse effects related to them [3]. The present study was conducted to revisit and rationalize local flaps used for nasal reconstruction based on the type of defects and evaluate their outcome by measuring patient satisfaction and QoL, using the FACE-Q appraisal scales. The FACE-Q scale was an important tool to use, so we attempted a linguistic validation of the scale by translating it into our local language (Hindi) after obtaining approval from the developers.

## Materials and methods

Materials and methods

A total of 23 patients were operated between September 2021 and December 2022 in the Department of Plastic Surgery and were included in the study. Informed, written consent was obtained from all the patients prior to their inclusion in the study. Permission for the study was sought from the Institutional Ethics Committee with approval number 2020/EC/1905, ECR/BHU/Inst/UP/2014/RE-registration-2017/dt.31.01.2017. Inclusion criteria comprised all patients presenting with single or multiple nasal defects, who were of either gender and consented to participate in the study. Patients suffering from any systemic illnesses and who didn’t give consent were excluded from the study. All data reporting was standardized according to the STROBE (STrengthening the Reporting of Observational Studies in Epidemiology) guidelines.

FACE-Q questionnaire

Permission to translate and use the FACE-Q module in the local language was obtained from the original authors (Appendix A). The FACE-Q questionnaire used comprised three modules: Satisfaction with Nose (10 questions), Satisfaction with Nostrils (5 questions), and Adverse effects with Nose (4 questions) (Appendix B). In the first two modules, patients were asked to rate their satisfaction/dissatisfaction pre- and postoperatively on a scale of 1 to 4, with 1 being ‘very dissatisfied’; 2 being ‘somewhat dissatisfied’; 3 being ‘somewhat satisfied’, and 4 being ‘very satisfied’. In the Adverse effects of Nose module, patients were asked about any problems they noticed with their nose before and after surgery and were asked to answer on a scale from 1 to 4, with 1 being ‘not at all’; 2 being ‘a little’; 3 being ‘moderately’, and 4 being ‘extremely’.

In each of the patients, the nasal subunit involved was identified and was further categorized depending on whether the defect was small (<1.5 cm), superficial (skin & subcutaneous tissue), large (>1.5 cm), deep (supportive framework or lining was missing), or composite (defect extends from the nose to the adjacent cheek and upper lip). An appropriate flap was planned for each patient, considering the size and the type of defect. All patients were asked to scale the satisfaction with their nose and nostrils, and any adverse effects they noted, prior to the surgery, using the Hindi translation of the FACE-Q modules. 

After the surgery, the patients were discharged on the second or third postoperative day. Procedures requiring a second stage were performed after three weeks. Regular follow-up was advised, every two weeks for the first month, followed by a bi-monthly follow-up for the next six months. At the end of six months, patients were asked to score their satisfaction with their nose and nostrils, and any adverse effects they noted after the surgery, using the Face-Q questionnaire. Any complications noted after the procedure were also noted. Pre- and postoperative clinical photographs were collected, and sociodemographic data were compiled.

Interpretation and analysis of outcome measurement was done using SPSS version 27.0 software (IBM Corp., Armonk, NY, US). All data were represented as mean and standard deviation. A p-value of <0.05 was considered significant. Internal consistency of the Face-Q scale was assessed by calculating Cronbach’s alpha with 95% confidence intervals. Values over 0.70 were considered to have acceptable internal consistency.

## Results

A total of 23 patients were included in the study, the male-to-female ratio was 2.28:1, with a mean age of 27.92 years. A total of 51 sub-units were reconstructed, amongst which the nasal tip sub-unit was most commonly involved (N=11, 21.6%). Other frequently involved sub-units were columella (N=10, 19.6%) and ala (N=10, 19.6%), followed by dorsum (N=8, 15.7%), soft triangles (N=7, 13.7%), and sidewalls (N=5, 9.8%). The most common cause of nasal defects was trauma (N=39, 76.4%), followed by tumor ablation (N=5, 9.8%), infection (N=4, 7.84%), and congenital causes (N=3, 5.88%). The nasal defects were of the large (N=19, 37.25%), small (N=5, 9.8%), superficial (N=6, 11.76%), deep (N=10, 19.6%), and composite defect types (N=11, 21.57%). The distribution of the defects according to subunits and their causes is given in Table 1.

**Table 1:** Distribution of defects according to subunit, cause, and type of defect All values have been represented as N (%). Null values have been represented as N.

Subunit	Cause of defect				Type of defect					
	Trauma	Infection	Tumor ablation	Congenital	Small	Superficial	Large	Deep	Composite	Total
Dorsum	5 (62.5%)	0	2 (25%)	1 (12.5%)	0	3 (37.5%)	3 (37.5%)	0	2 (25%)	8 (15.7%)
Nasal tip	9 (81.81%)	0	2 (18.18%)	0	0	1 (9.09%)	5 (45.45%)	3 (27.45%)	2 (18.18%)	11 (21.6%)
Sidewalls	4 (80%)	0	0	1 (20%)	0	1 (20%)	2 (40%)	1 (20%)	1 (20%)	5 (9.8%)
Alar rims	8 (80%)	1 (10%)	1 (10%)	0	0	0	5 (50%)	3 (30%)	2 (20%)	10 (19.6%)
Soft triangles	6 (85.71%)	1 (14.28%)	0	0	0	1 (14.28%)	3 (42.85%)	1 (14.28%)	2 (28.57%)	7 (13.7%)
Columella	7 (70%)	2 (20%)	0	1 (10%)	5 (50%)	0	1 (10%)	2 (20%)	2 (20%)	10 (19.6%)
Total	39 (76.47%)	4 (7.84%)	5 (9.80%)	3 (5.88%)	5 (9.80%)	6 (11.76%)	19 (37.25%)	10 (19.60%)	11 (21.57%)	51 (100%)

**Figure 1:**
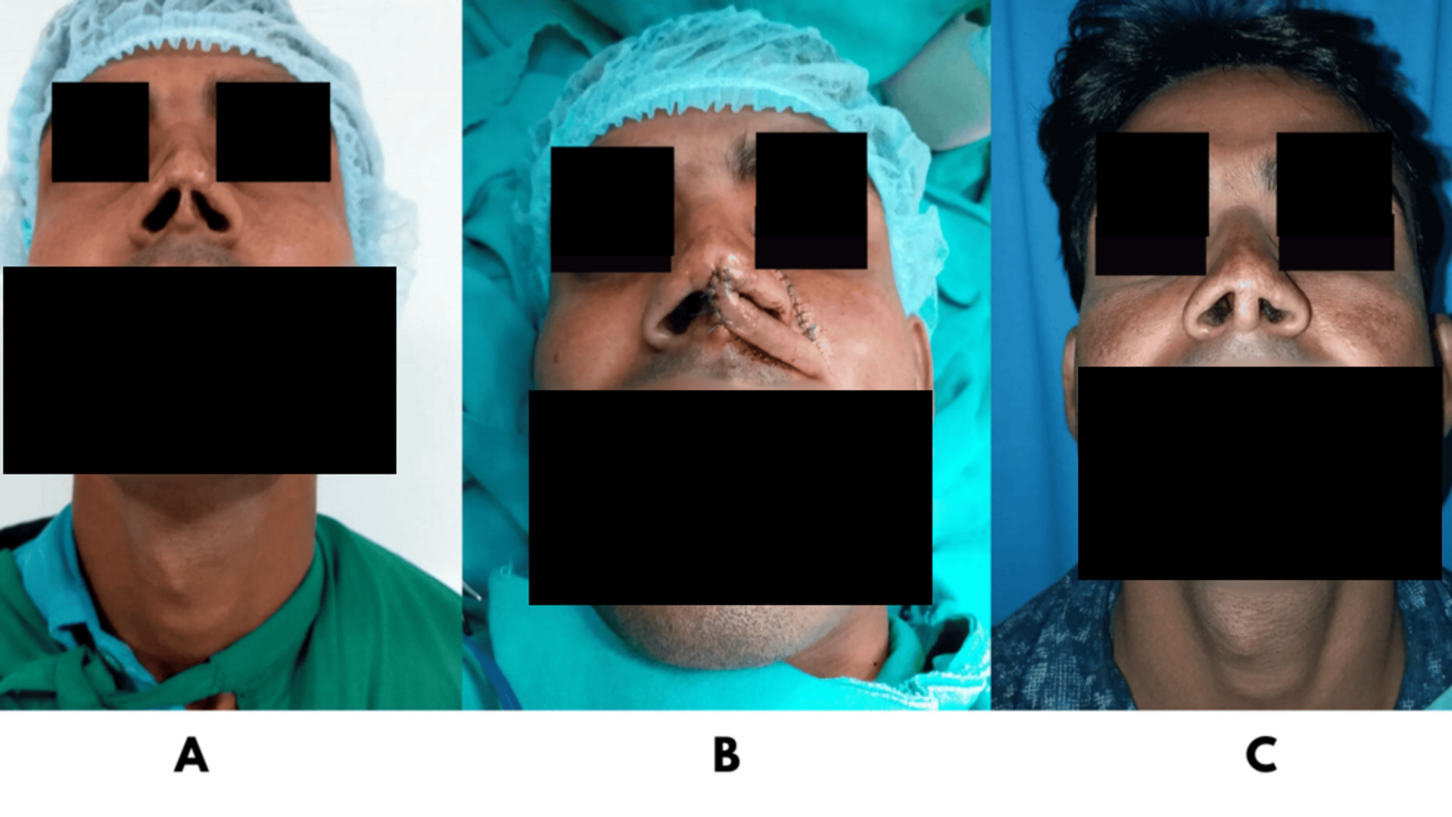
A: Preoperative image with columellar defect; B: Nasolabial flap used to reconstruct defect; C: Postoperative image with reconstructed columella

**Figure 2:**
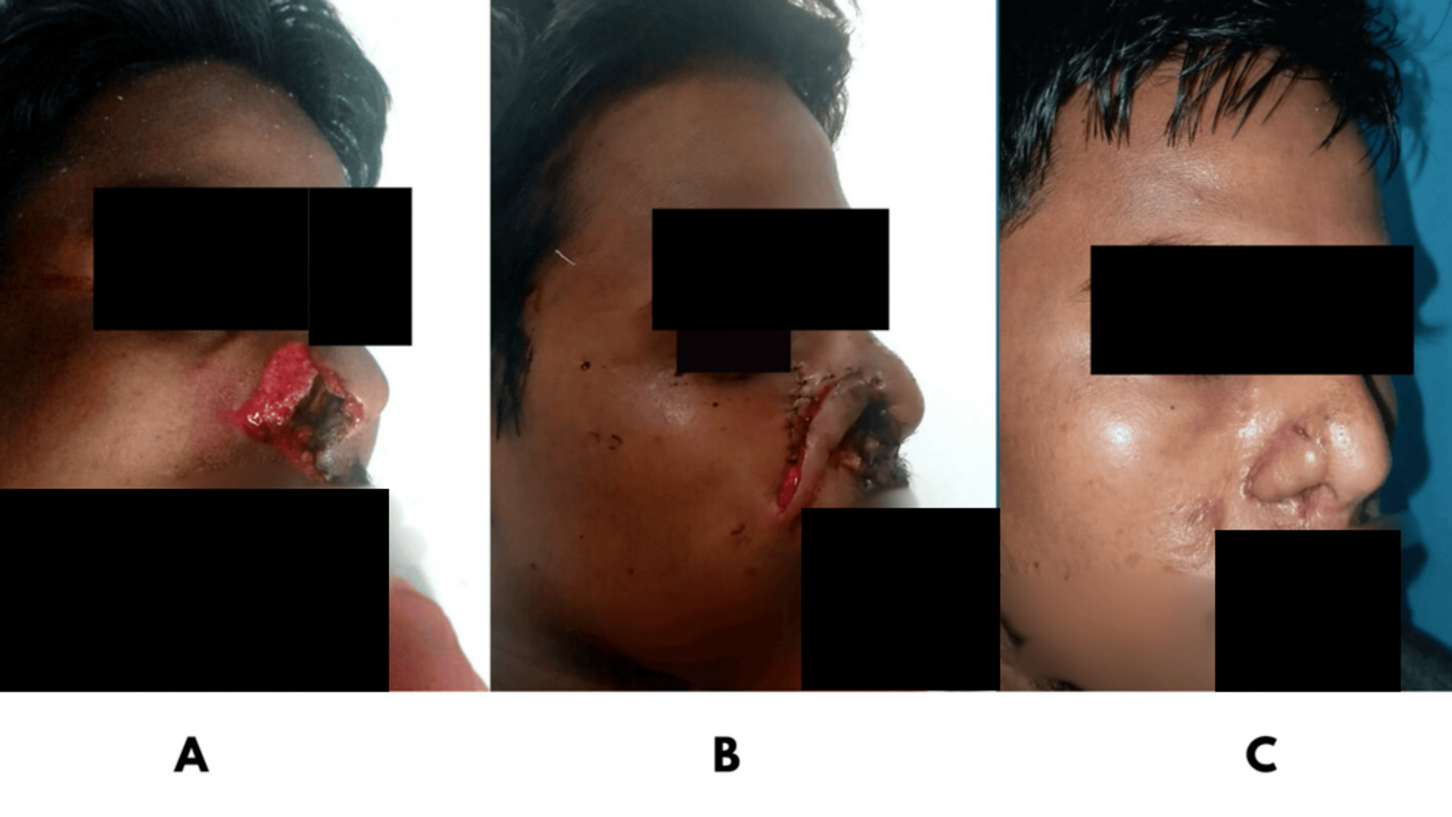
A: Preoperative image with defect of alar rims and sidewalls; B: Nasolabial flap used to reconstruct defect; C: Postoperative image with good alar rim contour

**Figure 3:**
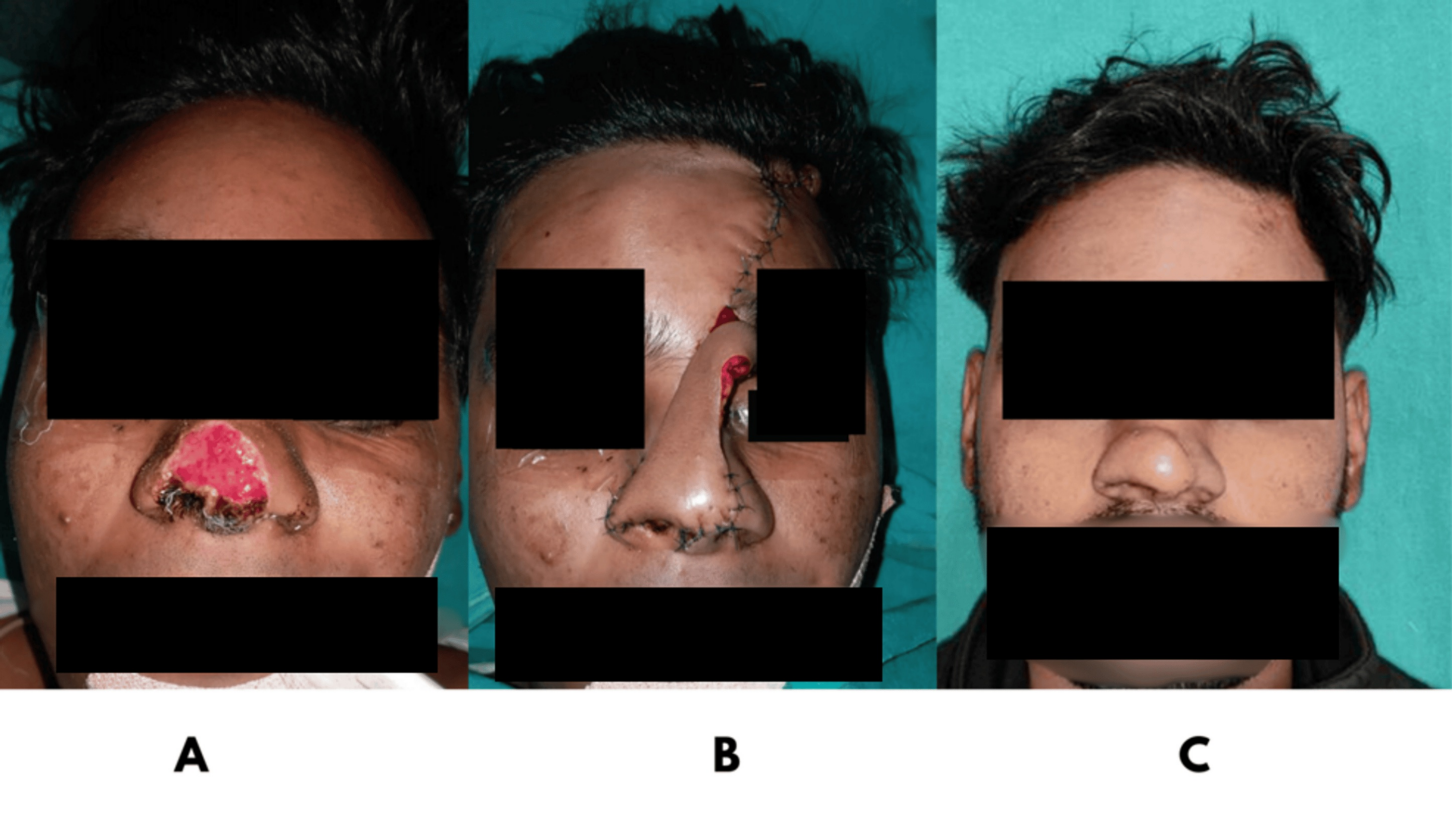
A: Preoperative image with defect of the nasal tip, dorsum, soft triangles, and alar rim; B: Paramedian forehead flap used to reconstruct defect; C: Postoperative image

**Figure 4:**
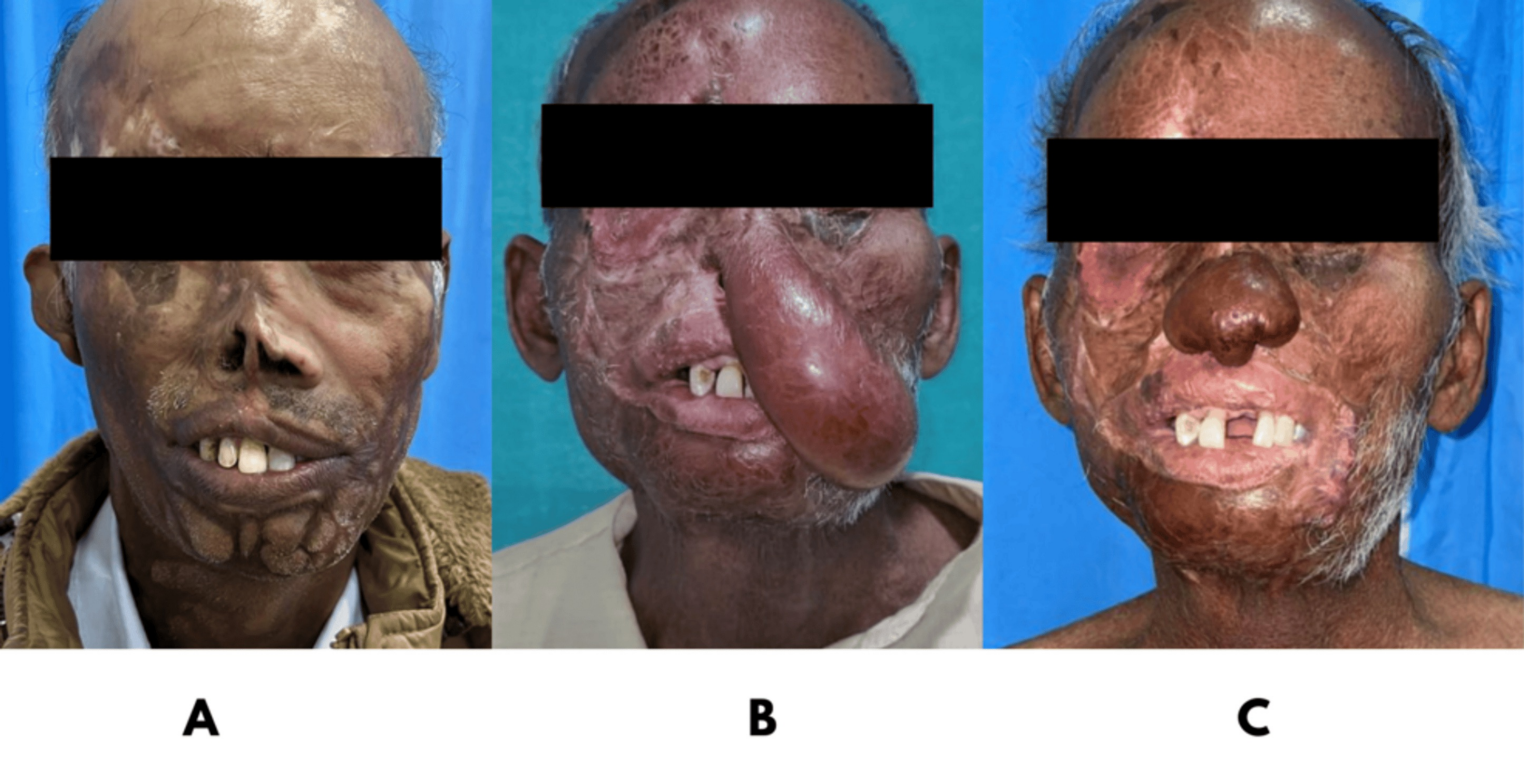
A: Preoperative image with a composite nasal defect after burns; B: Deltopectoral flap used to reconstruct defect; C: Postoperative image

The defects were resurfaced with a full-thickness skin graft in 4 (17.39%), V-Y advancement of local tissue in 2 (8.6%), deltopectoral flap in multiple stages in 1 (4.34%), axial frontonasal flap in 1 (4.34%), nasolabial flap in 6 (26.08%), and paramedian forehead flap in 9 (39.13%) cases, respectively (Table 2, Figures 1-4).

**Table 2:** Distribution of defects according to the method of nasal reconstruction used The number of defects is represented as N (%).

Method of reconstruction	Number of defects
Full-thickness skin graft	4 (17.39%)
V-Y advancement flap	2 (8.6%)
Deltopectoral flap	1 (4.34%)
Axial frontonasal flap	1 (4.34%)
Nasolabial flap	6 (26.08%)
Paramedian forehead flap	9 (39.13%)

The nasal framework was reconstructed with auricular and septal cartilage in 11 patients; 5 patients (21.73%) had large defects, 4 patients (17.39%) had deep defects, and 2 patients (8.69%) had a composite defect. The flaps used for reconstructing different types of defects were as follows: superficial (4 full-thickness graft), small nasal defects (nasolabial flap in 3 and V-Y advancement in 2), large nasal defect (paramedian forehead flap in 5, nasolabial flap in 1, axial frontonasal flap in 1), deep nasal defect (paramedian forehead flap in 3, nasolabial flap in 2), and the Composite nasal defect was managed with a deltopectoral flap in 1 and a paramedian forehead flap in 1. Lining was reconstructed with the folded skin of the paramedian forehead flap in 6 (26.08%), ipsilateral mucoperichondrial flap in 3 (13.04%), folded skin of the deltopectoral flap after thinning in 1 (4.34%), and local hinge flap in 1 (4.34%) case.

The outcome analysis after evaluation with the FACE-Q questionnaire pre- and postoperatively was as follows. The preoperative mean ± SD scores were: 21.26 ± 4.21 for satisfaction with nose, 12.3 ± 4.48 for satisfaction with nostrils, and 10.13 ± 2.26 for adverse effects noted in nose. The postoperative mean ± SD scores were: 32.4 ± 1.7 for satisfaction with nose, 16.04 ± 2.05 for satisfaction with nostrils, and 5.4 ± 0.65 for adverse effects noted in nose.

On comparison of the preoperative and postoperative scores, significant improvements were noted in satisfaction with nose (p<0.0001, degrees of freedom=23, t=14.73), and satisfaction with nostrils (p<0.0001, degrees of freedom=23, t=5.94) and a decrease in adverse effects of the nose (p<0.0001, degrees of freedom=23, t=11.2).

**Figure 5:**
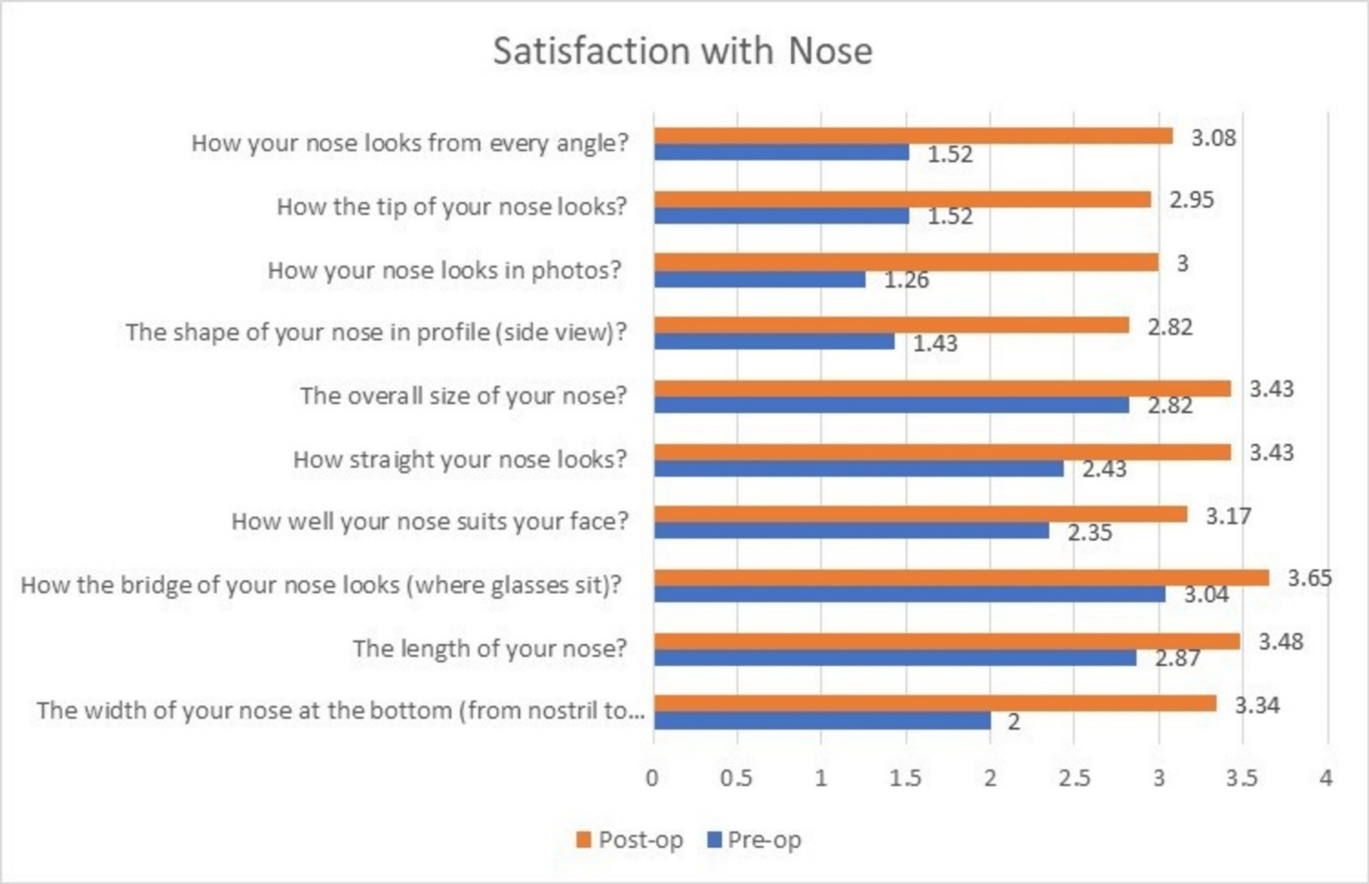
Change in mean scores of ‘Satisfaction with nose’ postoperatively

**Figure 6:**
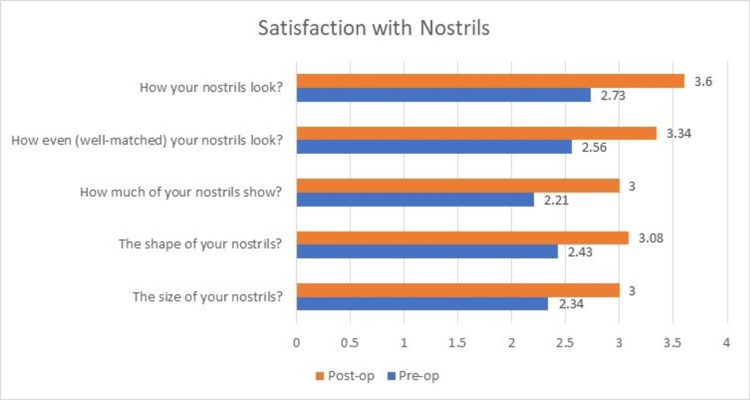
Change in mean scores of ‘Satisfaction with nostrils’ postoperatively

**Figure 7:**
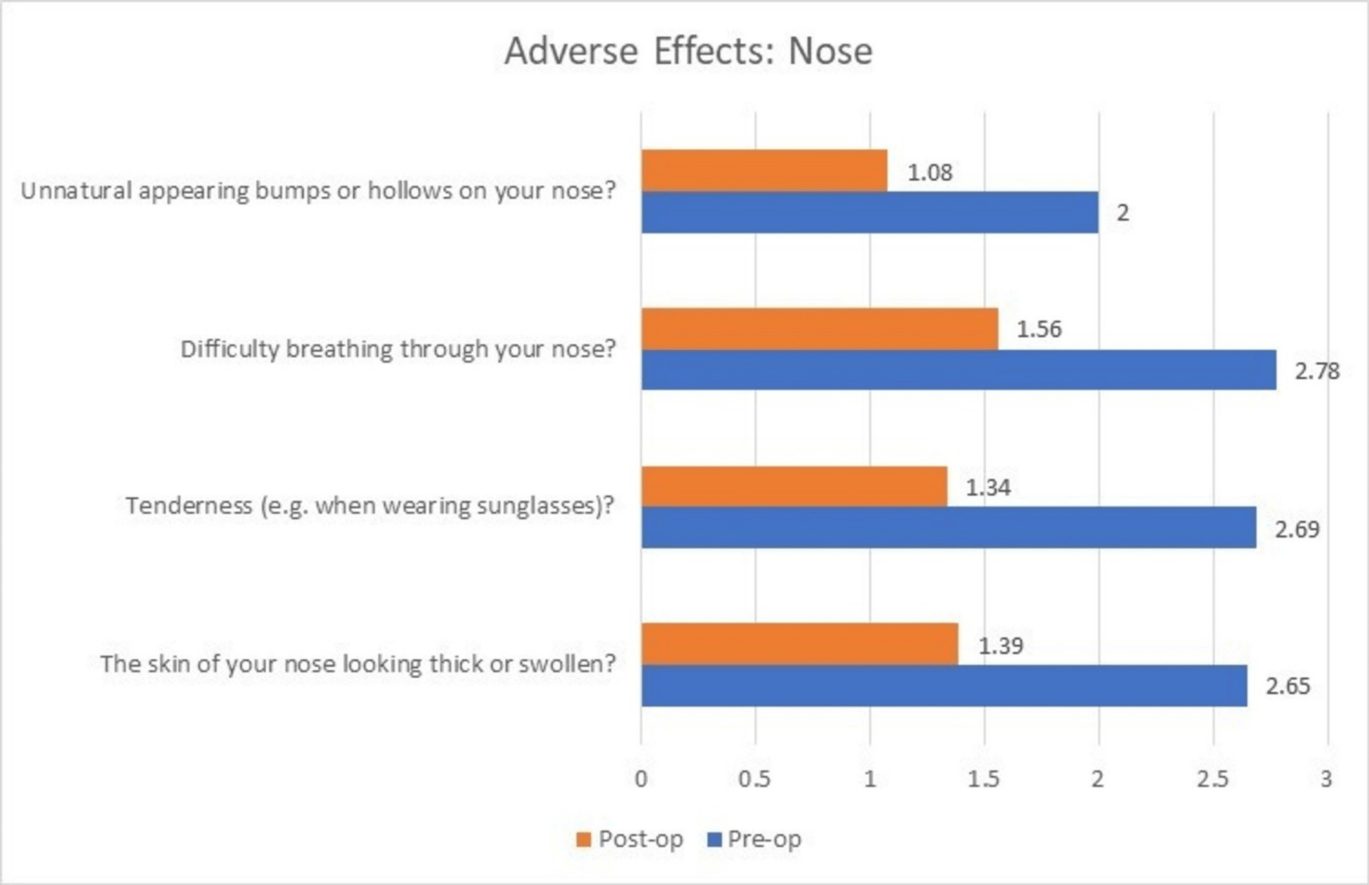
Change in mean scores of ‘Adverse effects: Nose’ postoperatively

All the patients showed significant improvements in their scores for satisfaction with nose, whereas three of the patients showed no improvement in further satisfaction with their nostrils postoperatively. Only 11 out of 23 patients showed significant improvement in their scores for satisfaction with nostrils owing to the significant amount of deformity in the structure of nostrils. Similar to the satisfaction with their nose, almost all the patients showed a drastic decrease in the adverse effects with their nose (Figures 5-7). The results showed a significant improvement in outcomes after using the reconstructive techniques outlined above for the respective defects.

## Discussion

Nasal reconstruction has evolved over time, to give satisfactory aesthetic results in patients suffering from nasal defects. The limited options for nasal reconstruction and the varied aetiologies contributing to different sizes and shapes of defects have resulted in no predefined consensus or guidelines regarding which flap option would be considered best for a specific defect [4]. For this purpose, our study has classified nasal defects as per current literature into five types: superficial, small, large, deep, and composite defects. 

Reconstruction of nasal defects was done using traditional nasal reconstruction techniques and their outcomes were evaluated. There has been a paucity of a reliable outcome measurement system for the evaluation of the reconstructed nose. Evaluation of patient satisfaction and QoL after cosmetic and reconstructive surgeries has become crucial today. Proper, quantifiable outcome analysis helps surgeons individualize reconstructive options catering to a specific type of defect. Questionnaires such as Rhinoplasty Outcome Evaluation, Nose Obstruction and Septoplasty Effectiveness Scale lack internal consistency and structural validity, as they don’t focus on the evaluation of different areas of the nose separately, and how they impact the QoL before and after surgery [5,6]. Hence, we took the help of the FACE-Q score developed by Klassen et al. and translated it into the Hindi language for its use in the Indian subcontinent after obtaining permission from its developers.

In our series, 23 patients with a nasal defect were re-surfaced, after judicious debridement of the defect margins. Most of the defects in the present study had been due to trauma. In cases of tumors over the nose, excision with free margins was followed by reconstruction, depending on the type of defect.

In the present study, full-thickness skin grafts from post-auricular skin were used to cover small defects (size < 1.5 cm^2^), which involved only the skin. Some of the similar skin-only defects involving the columella were treated with V-Y advancement flaps raised from the adjacent tissue. Full-thickness skin grafts were most preferred by the patients too, as they gave quick and safe coverage to the skin defect. However, color mismatch and contour irregularity were observed in two of the patients. McCluskey et al. have also agreed upon the feasibility of full-thickness skin grafts in covering superficial skin defects, however, they reported lower satisfaction scores with the outcomes owing to the contour irregularities and color mismatch noted in these cases [7]. Full-thickness skin grafts harvested from the forehead can give a better color match if the defect size is less than 1 cm. Also, setting the graft at an appropriate tension to the defect can avoid contour irregularities and hyperpigmentation that occur due to the contraction of the grafts in the postoperative period. For columellar defects less than half of its length, V-Y advancement was done by borrowing skin from the upper lip [8,9]. No deformities were noted in the upper lip by this method.

Cartilage support is integral to maintaining the nasal structure and functioning of the airway. Partial thickness defects that involve skin and cartilage warrant the use of autologous cartilage to restore the deficit in its framework. However, smaller cartilage defects that don’t cause any significant loss in support may be just covered with a local flap. The auricular concha and nasal septum are usually used for harvesting the cartilage for the nose. Auricular concha with its curvatures is a good option to reconstruct the alar portions of the nose. However, cartilage resorption and deformation have been noted with contracture of the covering skin flaps as time goes by. This accounts for the dissatisfaction with nostrils in 12 out of 23 patients in our study. Overall, the functional and aesthetic outcomes following a cartilage reconstruction have been good to excellent in various studies [10,11].

In full-thickness defects, it is imperative to give an inner lining for the proper look and function of the nose. Various techniques have been described to provide an inner lining, the simplest being the use of skin grafts. However, due to their lack of intrinsic blood supply, cartilage extrusion has been noted with their use; and hence, we avoided using that in our patients. Ipsilateral mucoperichondrial flaps based on the septal branch of the superior labial artery, as described by Burget and Menick, are often used to give an intranasal lining [12]. However, owing to their less reliable blood supply, their use along with cartilage grafts has been debatable. We used the ipsilateral mucoperichondrial flap in three of our patients. A folded paramedian flap has recently outgrown its significance in terms of providing inner lining. A thinned-out and folded paramedian flap has a reliable vascular supply and gives better results in providing the inner lining. This method has been used in six of our patients and the results have been excellent. A very small inner lining defect was observed in one of our patients, and a local hinge flap was used for lining. This did not give the nostril a very good appearance.

Combined partial and full thickness defects were observed in large, deep, and composite types of nasal defects. These defects were the most challenging ones to reconstruct. As they involve multiple subunits in different thicknesses, it is very important to choose an option that provides a wholesome coverage to the defects [13,14]. Also, different portions of the defect heal and contract in myriad ways, leading to contour and texture mismatch in such cases, which leads to multistage corrective surgeries in the late postoperative period [15,16]. We obtained satisfactory results using folded paramedian flaps and nasolabial flaps for lining and cover in these patients.

Recently, the whole trend of outcome measurement has shifted from the clinician’s perspective to the patient’s perspective. With that, there has been an upsurge in PRO tools to measure outcomes after surgery. The FACE-Q module developed by Klassen et al. is a reliable tool to measure PRO in different aesthetic units of the face after surgeries. We produced a Hindi-translated version of the Satisfaction with nose, Satisfaction with nostrils, and Adverse effects with nose questionnaires to use them in our study in the Indian subcontinent. On assessing the outcomes, we noticed an overall significant improvement in the FACE-Q scores postoperatively. In partial defects that were reconstructed with full-thickness skin grafts, we noticed better satisfaction among the patients, as it gave them a faster recovery. However, some cases reported dissatisfaction in later follow-ups owing to the color mismatch. In partial-thickness defects involving both the skin and cartilage and total-thickness defects, the paramedian forehead flap was noted to have the best results in all the satisfaction and adverse effects domains of the FACE-Q scale. This suggests that the paramedian forehead flap has stood the test of time in nasal reconstruction [17,18].

The use of PRO removes the surgeon’s bias toward a specific procedure, and guides us toward a better approach to the clinical problem in front of us, taking into consideration the patient’s satisfaction with the outcome instead. A limitation that we came across using FACE-Q is that it doesn’t take into consideration many factors like the duration and multiple stages of a surgery, which also indirectly affect the outcome and QoL in the patient.

## Conclusions

Nasal reconstruction, owing to its complex anatomy, has consistently been a topic of discussion. The paramedian forehead flap is the workhorse flap for simple and complex nasal defects. Comprehensive information about the outcome of nasal reconstruction can be measured using the FACE-Q scale, which is a patient-reported outcome assessment. This evaluation will help the surgeon in regular assessments and improvements of the outcome of surgery.

## Figures and Tables

**Figure 8:**
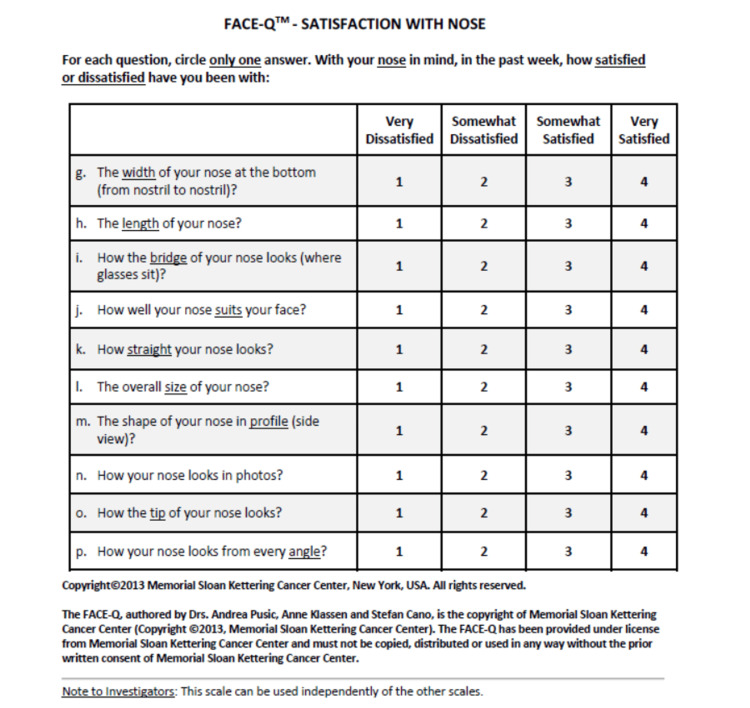
FACE-Q - satisfaction with nose Permission of use obtained

**Figure 9:**
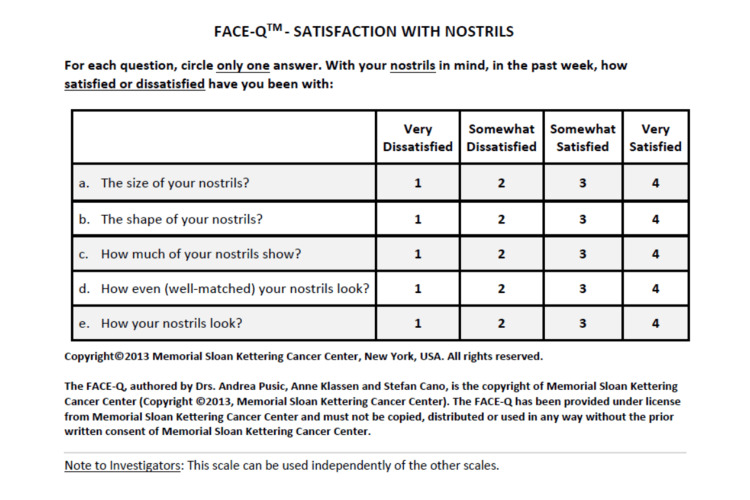
FACE-Q - satisfaction with nostrils Permission of use obtained

**Figure 10:**
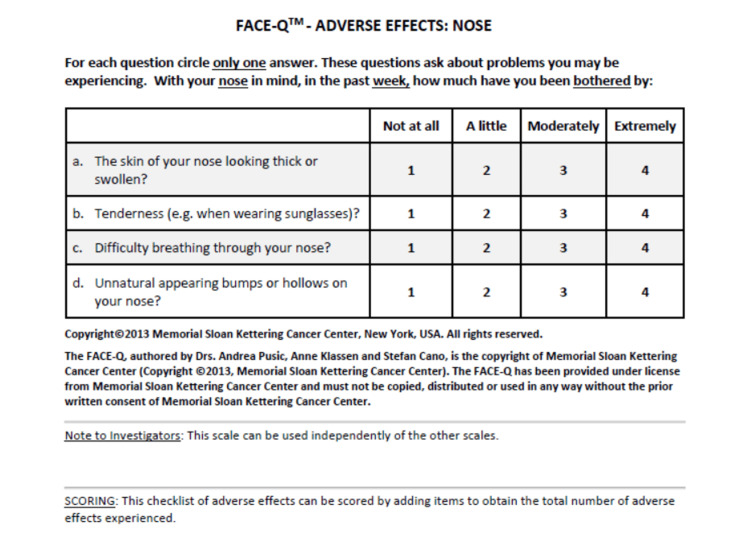
FACE-Q - adverse events with nose Permission of use obtained

## Appendices

Appendix A 

**Table 3:** Hindi translation of FACE-Q: Adverse effects - Nose Copyright©2013 Memorial Sloan Kettering Cancer Center, New York, USA. All rights reserved. The FACE-Q, authored by Drs. Andrea Pusic, Anne Klassen, and Stefan Cano, is the copyright of Memorial Sloan Kettering Cancer Center (Copyright ©2013, Memorial Sloan Kettering Cancer Center). The FACE-Q has been provided under license from Memorial Sloan Kettering Cancer Center and must not be copied, distributed or used in any way without the prior written consent of Memorial Sloan Kettering Cancer Center.

	हर्गिज नहीं	थोड़ा सा	परिमित रुपसे	अत्यंत रुपसे
आपकी नाक की त्वचा मोटी या सूजी हुई लग रही है?	1	2	3	4
दर्द (जैसे धूप का चश्मा पहनते समय)?	1	2	3	4
आपकी नाक से सांस लेने में कठिनाई?	1	2	3	4
आपकी नाक पर अस्वाभाविक दिखने वाले कोई सूजन या गड्ढे?	1	2	3	4

Appendix B 
